# Shifting your research from X to Mastodon? Here’s what you need to know

**DOI:** 10.1016/j.patter.2023.100914

**Published:** 2024-01-12

**Authors:** Roel Roscam Abbing, Robert W. Gehl

**Affiliations:** 1 Malmö University, Malmö, Sweden; 2 York University, Toronto, ON, Canada

## Abstract

Since Elon Musk’s purchase of Twitter/X and subsequent changes to that platform, computational social science researchers may be considering shifting their research programs to Mastodon and the fediverse. This article sounds several notes of caution about such a shift. We explain key differences between the fediverse and X, ultimately arguing that research must be with the fediverse, not on it.

## Main text

### Introduction

Since October 2022, when Elon Musk finalized his purchase of Twitter, hundreds of thousands of people stopped using Twitter in favor of Mastodon. Developed in 2016, Mastodon has positioned itself as a Twitter alternative, providing a relatively soft landing for people leaving Twitter. As Musk continues to do some baffling things with Twitter—ranging from firing thousands of employees to openly insulting advertisers to changing its name to “X”—waves of people keep shifting from Twitter/X to Mastodon and a broader network called the “fediverse.”

It is not only users who are shifting from Twitter to Mastodon—researchers are considering making the shift as well. Changes to Twitter/X’s application programming interface (API), coupled with its decreasing userbase and increasingly poor reputation, have prompted researchers to rethink social media research. While Mastodon has positioned itself as a Twitter alternative, it is actually quite different from Twitter, not only for users but also for researchers.

We have been using, studying, and operating Mastodon servers for the past 7 years. Between us, we have published peer-reviewed research about Mastodon and are in the process of writing lengthy studies of it (in the form of a dissertation and a book). We also have extensive experience running and moderating Mastodon instances.

Drawing on our experience, this paper discusses computational research ethics on Mastodon. We raise four specific issues researchers should consider before shifting their research programs from Twitter/X to Mastodon. Mastodon is not one distinct site; it is operated by a plurality of actors that exists in a larger ecosystem of alternative social media platforms known as the fediverse. While Mastodon might appear to be a stand-in replacement to Twitter/X, its structure and culture demands that researchers change their approaches to research ethics when it comes to consent and data collection. The privacy expectations of Mastodon users are quite different, and this affects how a researcher should use the Mastodon API. In addition, recruitment techniques that worked in corporate social media will not work on Mastodon and the fediverse.

We conclude with three recommendations. First, we suggest studying instances, not users. Similarly, we argue that researchers should enroll instance admins as research partners. Finally, we call for parsimony among fediverse researchers.

Ultimately, while the fediverse is unique and requires adjustments for researchers, there can be rewards. The fediverse is not only an opportunity for social networking on different terms—it can also be seen as a new beginning for social network research, part of a new relationship between academia and its research subject. Instead of research conducted on social data, we can do research with, and for the benefit of, social groups.

### Mastodon and the diverse fediverse ecosystem

At first glance, Mastodon appears to replicate much of Twitter/X: it is a microblogging system with follower/followed relationships, hashtags, and an open API similar to Twitter’s. Using this API, application developers and researchers alike can computationally query the network to retrieve accounts, posts, and more. Libraries for different programming languages already exist to make interfacing with the API easier. In principle, this allows researchers to directly migrate their research practices from Twitter to Mastodon.

There are also important differences, however, having to do with structure and culture. Mastodon is a federated social platform, which means that the social network of Mastodon is not operated and maintained by a single party but rather by a plurality of actors. This includes not only Mastodon (the project that makes the software, found at https://joinmastodon.org) but also thousands of Mastodon “instances” (distinct installations of the Mastodon software). There is also a range of different platforms that use the ActivityPub protocol, making up a larger network of social platforms known as the “fediverse.” Both Mastodon and the broader fediverse are culturally very distinct from Twitter, which raises major implications for research ethics.

Like Mastodon, the broader fediverse social network is made up of distinct instances that interconnect. Users sign up to specific instances, and instances host the user data. These instances are operated by actors ranging from the Mastodon project itself to individual under-resourced volunteers, democratic associations, public institutions, for-profit corporations, academic associations, and groups of friends.

While each of these instances runs similar software, they tend to operate under different governance structures and expectations of use. This is reflected in the diversity of documents that regulate user conduct, such as the “terms of service” or “code of conduct” documents. Some of these instances describe themselves in very general terms, but there are many that are run and used specifically by historically marginalized populations, such as queer folks, Black folks, or Indigenous people. A cursory look at the Mastodon project’s server picker under the categories “LGBTQ+,” “activism,” and “furry” will reveal as much, especially given that the instances listed there applied to be included on that page.

At the same time, fediverse software packages themselves can be very diverse in terms of how they look, what media they support, and what features they have. To give some examples; Peertube (https://joinpeertube.org) is oriented around sharing videos and livestreams. Pixelfed (https://pixelfed.org/how-to-join) is based on images. WriteFreely (https://writefreely.org/) and Wordpress are oriented around long-form text. Besides Mastodon, there are other microblogging systems on the fediverse, such as Pleroma (https://pleroma.social/), Misskey (https://join.misskey.page), and GoToSocial (https://gotosocial.org/). They all have different interfaces, features, and culturally diverse userbases and development teams.

Accessing the API of a fediverse instance can, in theory, retrieve material from any of these diverse instances. And this is where fediverse research is distinct from Twitter/X research. While data culled from the Twitter API would be governed by a single terms of service agreement, and while Twitter users are all collected together in one site, the fediverse is comprised of tens of thousands of distinct sites. When accessed through the Mastodon API, material from all these different applications and contexts can be flattened together. Because of the different interfaces and cultures of use and expectations, users can be surprised by how their data circulates through the network and what it looks like on other software. In addition, use cultures vary across software packages: analysis of data from Pleroma, for example, is not likely to shed much light on Mastodon or Pixelfed. Researchers should thus be careful not to make claims about the whole network based on data from only one type of fediverse platform.

### Expectations of privacy

Because Mastodon and the fediverse are comprised of thousands of distinct instances, each with their own culture, norms, and explicit terms of service, researchers need to consider the various ways privacy is conceived across the fediverse. According to the Association of Internet Researchers (AoIR) Internet Research Ethics guidelines, “different platforms have different use cultures that lead to different ethical implications.”^1^ For example, a blogging platform, whose authors conceive of their work as being a publication, requires less privacy considerations than, to take another example, a small forum dedicated to discussing health matters.^2^ And of course, distinct legal environments (say, Canada versus the US versus the EU) have different regulations about online data privacy.

For those researchers moving their programs from Twitter/X to Mastodon and the fediverse, first consider the distinct conceptions of privacy found on those two platforms. Twitter grew out of a mentality of “Web 2.0” and openness, which meant that there was a strong focus on the open accessibility of the content. The majority of posted material has historically been visible on the web and indexable by search engines as well as accessible through the open API. Over its history, Twitter’s different CEOs continuously referred to the platform as a public town square.

Taking many design queues both from Twitter and earlier federated social web projects, Mastodon is arguably based around much of the same ideas and assumptions—perhaps even more so, since Mastodon’s code is open source. Early in Mastodon’s history, however, the project attracted many queer and trans users and developers who shifted the design of the project to explicitly include more privacy and safety features. Some of the earliest Mastodon instances, such as awoo.space, are conceived of as very private spaces. Ever since, a tension has existed between the Mastodon project’s commitment to openness on the one hand and the users’ and developers’ focus on safety and privacy on the other.

The diversity of views on privacy on the fediverse means that there is no “one stop shop” for informed consent. The existence of a Mastodon API is not to be confused with the consent of Mastodon users to have their data included in studies. Instead, each instance has norms or explicit rules about how researchers should approach user data. More generally, privacy is often poorly understood when reduced to dichotomies between data that are public and private or sensitive and non-sensitive. Rather, as Helen Nissenbaum argues, privacy is best understood as information flows in specific contexts, that are governed by norms that decide whether a flow is appropriate or not.^3^ When the integrity of the specific context is breached, this becomes a privacy violation. In other words, the same data that someone might approve of sharing in an alternative social network might be seen as a violation of their privacy when it unexpectedly emerges as part of a study or a search result. Regardless of whether that data is technically public or not, expectations should be an overriding consideration. Thus, while a post may be labeled “public” in the Mastodon API, the user who posted it did not expect it to be taken out of the social media context and repurposed. We argue researchers should be aware of these contradictions and not take advantage of them but rather try to understand user privacy expectations on a case-by-case basis.

### Histories of poor social media research

So, the story so far: there is a diversity of cultures across the fediverse. And yet, across the network, there seems to be a strong desire for privacy among fediverse users; fediverse users desire not to be monitored, included in automated research, or have their posts included in search engines without their explicit consent. In part, these desires reflect the fediverse’s distaste for corporate social media, particularly in light of unethical uses of personal data, such as the Cambridge Analytica scandal and the Facebook emotional contagion study.^4^^,^^5^

Because of these histories, fediverse users are very savvy when it comes to identifying and critiquing poorly conducted research studies on the fediverse. For example, in 2019 researchers published a paper called “Mastodon Content Warnings: Inappropriate Contents on a Microblogging Platform” that studied the use of the “content warning” feature of Mastodon, where users can add a text describing the nature of their post.^6^ The study relied on the collection of a large sample of posts that had been accessed through instance APIs and was released as part of the paper. After reading the study, members of the fediverse, both technically savvy and academically trained, wrote a scathing open letter to the university arguing for the retraction of the study based on several flaws with the study.^7^ Both the dataset and the paper have since been retracted.

It is important to note the various types of mistakes identified by the open letter. The first involves ethical issues surrounding consent and data collection. The authors of “Mastodon Content Warnings” assumed not only that what is on the API is fair game but also that there would be no differences between fediverse instances’ various terms of service. Second, the “Content Warnings” paper made the procedural mistakes of improperly deanonymizing that data before publishing it. Finally, the paper had analytical shortcomings grounded in a failure to recognize the range of uses that the “content warning” feature has in practice. The API itself can be misleading here, as posts retrieved via the API contain a flag called “sensitive: true” or “sensitive: false.” The feature is used by authors to provide a summary of material being shared and to require a reader to perform an additional click to display the material. This gives readers the chance to decide whether they engage with the material or not. The feature has not only been used to indicate that something might be sensitive to readers, however; it has also been used as part of a joke or to protect the reader from movie spoilers. As the authors of the open letter state, “Such CWs [content warnings] are acts of courtesy, not signals of ‘inappropriate’ content.” This is an observation that would have emerged relatively quickly from a user-centric study but is easier to miss with an API-centric research focus.

This is not an isolated instance. We have written critically before about another paper in which the authors collected posts with images and ran them through a cloud-based service to determine whether they were explicit.^8^^,^^9^ The authors failed to get consent from Mastodon users for inclusion in the study or for uploading their data to such a service. In addition, the paper makes claims about Mastodon, but a significant amount of the data originated from a Pleroma instance. The paper also makes claims about weak moderation on the fediverse, since many images the authors culled included sexual content. This claim is false; some instances are extremely active in moderating against sexual content, while others allow it with content warnings. Again, the fediverse contains a diversity of approaches, so making a blanket claim about poor moderation does not hold up.

While we describe issues particular to Mastodon, this only drives our main point: other software packages in the fediverse, such as Pleroma, PeerTube, or Pixelfed, have their own histories and thus conceptions of privacy. In addition, different software can represent similar data in different ways. Diligent researchers should thus account for different expectations of privacy and consent between instances and projects. But a more general point can be made about the Mastodon API; it tends to flatten these differences between instances and software packages and it obscures the role of interfaces altogether. We would thus argue that because of this artificial flattening, researchers cannot make grand claims about the fediverse as a whole.

### Different approaches to recruitment

One major distinction between Mastodon and Twitter/X is that Mastodon (and much of the fediverse) does not use algorithms to shape timelines, nor do most instances gather personal information in order to sell it to marketers and advertisers. This is in reaction to surveillance capitalism, where users of free services such as Facebook, Instagram, or Twitter/X actually pay a price in the form of personal data. In surveillance capitalism, users exchange their data for access to their friends, and the platforms sell that data to marketers, who pay to place advertisements in user timelines. The platforms also use algorithms to feed specific, interest-based content—including such advertisements—to users.

In contrast, the vast majority of Mastodon instances do not collect personal data, and even if they did, the federated structure of the network would make it extremely difficult for a marketer to place advertisements in front of targeted users. Even if a marketer were to place such an ad, the rest of the network would quickly block it from spreading. In addition, all Mastodon posts are in chronological order. Instead of being shown out of chronological order by an algorithm, posts that are deemed valuable by Mastodon users are boosted by them, essentially being repeated in the chronological timeline.

This has an implication for research recruitment. Consider the common practice of paying to promote a study (e.g., advertising the existence of a survey).^10^ On the fediverse, such a practice would be extremely difficult: the researchers would have to contract with each instance to pay for a site-wide announcement. Given the norms of the fediverse, such an act would receive a great deal of resistance. Instead, researchers looking to promote surveys or recruit participants would have to join an instance, share a link to their survey, and hope that the rest of the fediverse boosts their post. For that to be effective, it is likely the research team will have to participate in the fediverse for some time, building up good will and a following.

### Recommendations

In light of the differences between X and Mastodon, we have several recommendations for researchers. First, however, we should point out our own positionality as qualitative researchers. Many of the issues we have highlighted are existing tensions between qualitative and quantitative approaches to research. This is not to say we are against quantitative research; to the contrary, we believe it can provide valuable insights into an emerging ecosystem. As we have shown, however, transposing practices from one environment to another without taking note of the particularities of the new environment runs the risk of drawing questionable conclusions or alienating the community of users. We believe our recommendations can help avoid these issues.

*Our first recommendation is to consider studying instances,not individuals.* This is broadly in line with the current and accepted privacy model of the fediverse, where the instance is the front door. This approach helps respect the privacy of individuals while still producing valuable knowledge. Currently, independent and verifiable usage statistics and trends about fediverse instances, software packages, and user numbers are sorely needed. Such work would be a valuable contribution. To date, fediverse researchers have needed to rely on resources with unclear methodologies and inclusion criteria, which are provided and maintained by volunteers. Consequently, these resources have tended to disappear or be unreliable. This work would provide insights into the social structure of the fediverse without violating user privacy.

Even when using the instance as the locus of a study, it is good to consider that differences exist between them. For example, there are differences between instance servers in terms of computational power, and smaller ones can disproportionally be impacted by the network traffic generated by data collection. Researchers should be aware of the burden crawlers and API requests may have on smaller instances.

*Our second recommendation is to work with instance admins, moderators, and the community at large to discover mutual research interests.* Much of the research conducted on Twitter/X is research on social practices. Instead of taking this approach, we recommend working with instance admins and moderators to discover their concerns and deliver research-based solutions. For example, we have previously argued that computational research on fediverse should initially focus on building the tooling to respect consent by engaging with the community.^11^ Mastodon’s privacy flags allowing users to opt-in to search engines could be a model for that.

Engaging with the community will make your own work easier in the long run because you will build partnerships with the communities you’re examining, answering research questions they find relevant. Unlike research on Facebook or Twitter/X, which can easily be ignored by those large corporations, research with the fediverse can have a major impact on the future of the network.

*Our third recommendation is to use the principle of parsimony.* As we have discussed, understanding the Fediverse solely through an API makes it easier to miss the subtextual and intertextual. This has not only consequences for the conclusions that are drawn from the data, but also for research ethics. Data drawn from particular software packages, instances or communities say something about those communities, but the parts do not always speak for the whole. An analogy would be to study one particular website to make claims about the world wide web as a whole. We believe this specificity can be achieved on the one hand by taking into account the ways that the API flattens the diversity on the one hand and drawing from multidisciplinary insights on the other.

While Mastodon and the fediverse are quite distinct from Twitter/X and other corporate social media, and while these systems present new challenges to researchers, the benefits for both researchers and for the fediverse can be tremendous. If researchers work with instance admins to produce useful knowledge, that work can be adopted by the fediverse, helping to improve a rapidly growing network. Instead of a situation where researchers must adapt to the changing whims of indifferent corporations, who might alter their APIs or terms of service at any moment, researchers who partner with the fediverse can develop long-term, reliable relationships that can last for entire careers.
